# The capture of circulating tumor cells by Labyrinth system as a tool for early stage lung cancer detection

**DOI:** 10.3389/fonc.2024.1474015

**Published:** 2024-10-30

**Authors:** Peipei Jin, Hong Li, Mingran Xie, Jie Tang, Siming Zou, Ruiting Wang, Bin Yu, Tao Chen, Ju Zhang

**Affiliations:** ^1^ Department of Clinical Laboratory, The First Affiliated Hospital of University of Science and Technology of China, Division of Life Sciences and Medicine, University of Science and Technology of China, Hefei, China; ^2^ Core Unit of National Clinical Research Center for Laboratory Medicine, Hefei, China; ^3^ Department of Research and Discovery, Suzhou Labyrinth Biotech Co., Ltd, Suzhou, China; ^4^ Department of Thoracic Surgery, The First Affiliated Hospital of University of Science and Technology of China, Division of Life Sciences and Medicine, University of Science and Technology of China, Hefei, China; ^5^ Analytical General Department, Coherus BioSciences, Camarillo, CA, United States

**Keywords:** Labyrinth system, circulating tumor cells, lung nodules, lung cancer, microfluidic chip

## Abstract

**Objectives:**

We focus on utilizing the Labyrinth system for the detection of circulating tumor cells (CTCs) in patients with lung nodules. Our aim is to evaluate CTCs isolated through the Labyrinth system as a biomarker for early-stage lung cancer (LC) detection.

**Methods:**

167 patients with low dose computed tomography (LDCT) diagnostic results for lung nodules and 31 healthy volunteers (HV) were enrolled. Blood samples were processed for CTC detection. LDCT positive (LDCT+) patients underwent surgery and were categorized into those with LC and those with benign lung diseases (BLD) based on their biopsy results. BLD Patients, LDCT negative (LDCT-) patients and HV served as controls. The correlation of CTC counts with LC, BLD, LDCT- and HV was investigated. Receiver operating characteristic (ROC) curves were used to assess the Labyrinth system’s diagnostic potential for early-stage LC.

**Results:**

Median CTC counts for LC, BLD, LDCT- and HV were 2.7 CTC/mL, 0.6 CTC/mL, 0.4 CTC/mL, 0 CTC/mL, respectively. Statistical analysis indicated CTC counts could distinguish LC from BLD, LDCT- and HV (p-Values < 0.001). Using a cut-off of 1 CTC/mL, the study showed 84.4% sensitivity and 82.4% specificity for LDCT+ patients. Specificity increased to 85.9% for patients with lung nodules and 88.2% for all participants. In conclusion, CTCs detected by the Labyrinth system can serve as a biomarker for early-stage LC detection for patients with lung nodules.

**Conclusions:**

CTCs identified by the Labyrinth system are a promising biomarker for early-stage LC detection in clinical practice.

## 1 Introduction

LC, the leading malignancy worldwide in terms of both incidence and mortality, has emerged as a major health concern demanding immediate attention. The high prevalence and impact of LC underscore the urgent need for effective prevention, early detection, and treatment strategies to address this formidable public health challenge (1, 2). Early detection of LC through the identification and evaluation of lung nodules with diagnostic methods such as chest X-ray and computed tomography is crucial for improving outcomes (3, 4). Tumor markers like carcinoembryonic antigen and neuron-specific enolase from blood samples offer more accessible and cost-effective ways for LC screening. However, those makers currently lack sufficient sensitivity and specificity for reliable LC screening and diagnosis in clinical practice (5, 6).

In recent years, CTCs obtained from blood samples have emerged as a promising and alternative method for LC diagnosis (7). CTCs, which detach from primary or metastatic tumors and enter the bloodstream, can be detected and analyzed through a “liquid biopsy” approach. CTCs detection shows promise in both identifying malignant lesions and monitoring disease progression in various solid tumors, including LC (8–11). Studies have indicated the presence of CTCs even at early-stages LC when conventional imaging techniques may fail to detect tumors (12).

Several studies have reported using CTCs in early-stage LC diagnosis or early detection. The CellSearch system, which isolates CTCs by binding to the epithelial cell adhesion molecule (EpCAM), only detected CTCs in 31% of LC patients (13). Meanwhile, the Isolation by Size of Epithelial Tumor Cells (ISET) method demonstrated higher sensitivity in detecting CTCs, reaching 50% in patients with non-small cell lung cancer (NSCLC) (14). Another method involving detecting CTCs by folate receptor, followed by a ligand PCR-based approach for diagnosis, has shown of 82% sensitivity in stage I–IV NSCLC and 82% sensitivity in LC including small cell lung cancer (15, 16). The selection of CTCs isolation techniques employed in studies of LC can significantly influence the clinical outcomes.

Labyrinth system is a label free microfluidic method for isolating CTCs, developed using a specially designed microfluidic chip by Dr. Nagrath’s group at the University of Michigan in 2017 (17). It utilizes the combination of long loops and sharp corners from the chip to generate inertial forces that focus CTCs and smaller white blood cells into separate outlets lines based on cell size. It has also been reported to enhance CTCs capture efficiency and numbers from hepatocellular carcinoma, pancreatic cancer and LC patients (18–20).

While previous studies used laboratory produced chips, this study at the First Affiliated Hospital of USTC in Anhui, China, employed industry-manufactured, single-use Labyrinth microfluidic chips produced by Suzhou Labyrinth Biotech. The study aimed to assess the potential of CTCs isolated by the Labyrinth system as a biomarker for detecting and diagnosing LC in patients with lung nodules identified through LDCT. Blood samples from enrolled participants were collected for CTCs. Patients exhibiting ground-glass opacity nodules identified through LDCT scans as LDCT+ were subsequently referred for surgery to acquire biopsies for pathological diagnosis. In this study, CTCs diagnosis results indicate an 84.4% sensitivity and 82.4% specificity for patients undergoing surgery, 84.4% sensitivity and 85.9% specificity for all patients with lung nodules, as well as 84.4% sensitivity and 88.2% specificity for all participants. These results highlight the potential benefits of employing CTCs identified by Labyrinth system for detecting and diagnosing early-stage LC, marking a significant advancement for clinical practice.

## 2 Materials and methods

### 2.1 Study design

**Figure 1:**
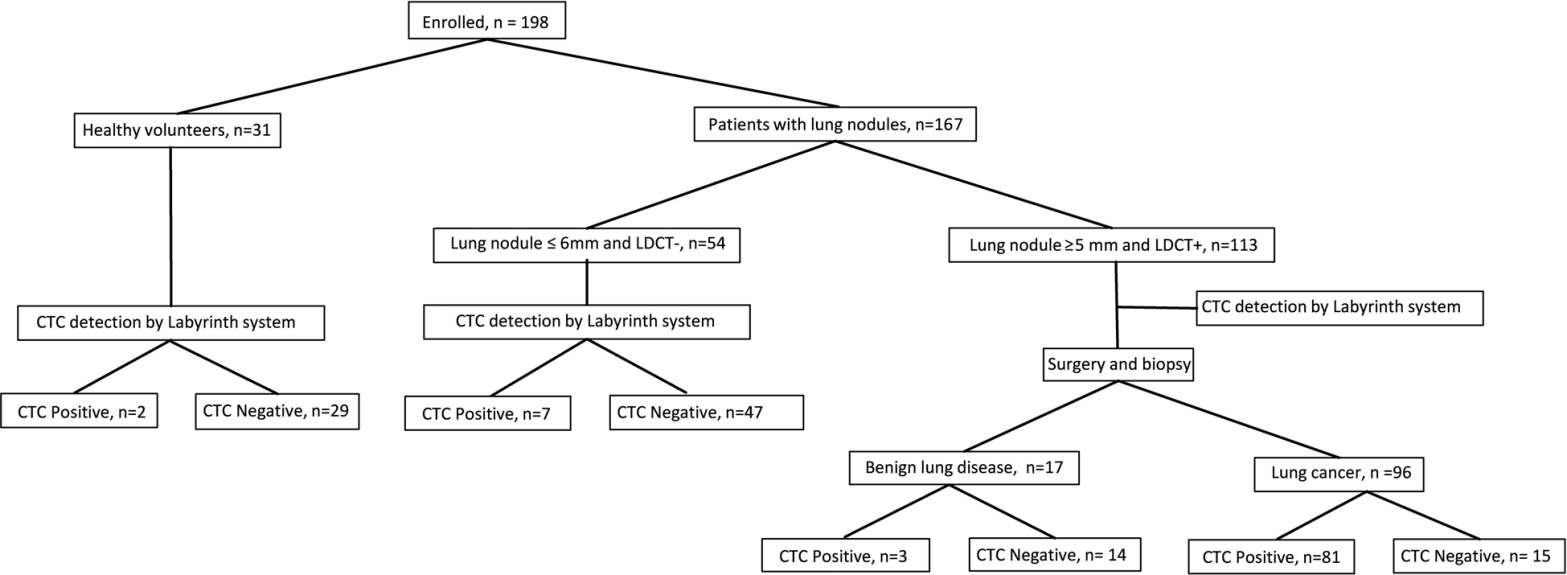
Clinical procedure flow chart for CTC detection by Labyrinth system.

A total of 198 individuals participated in this one center, prospective clinical study in the First Affiliated Hospital of USTC. Among them, 167 patients were diagnosed with lung nodules by LDCT. Out of these, 113 patients exhibited lung nodules larger than 5mm and received preliminary positive diagnostic outcomes from LDCT (appeared as a part-solid nodule with short spiculation, lobulation, pleural traction and pleural indentation), leading to their progression to surgical procedures. Lung tissues containing the nodules were meticulously extracted for biopsy and pathological diagnosis. Blood samples were obtained from these patients just before the surgery. Additionally, the remaining 54 patients with lung nodules smaller than 6mm and negative diagnostic outcomes from LDCT were only recruited for their blood samples on the same day as their LDCT examination. Furthermore, 31 healthy individuals were recruited. 10 mL blood samples were collected from all participants and shipped the same day to Suzhou Labyrinth Biotech Inc. with overnight delivery. Labyrinth system was applied for CTCs isolation, detection and identification. A flow chart describing the procedures was shown in **Figure 1**.

The basic information such as gender, age, LDCT diagnosis results (including lung nodule size and locations), and pathological diagnosis results for surgical patients were also collected. Approval for this study was granted by the medical ethics committee of the First Affiliated Hospital of USTC on June 2, 2022, with reference number 2022-ky062. This research adhered to the principles outlined in the Declaration of Helsinki. All participants were given informed consent and their privacy rights were always observed.

### 2.2 Pathological diagnosis

Surgically removed lung tissue was fixed in 10% neutral buffered formalin, processed routinely by dehydrating through graded alcohols, embedded in paraffin wax, sectioned (5 μm) and mounted on Superfrost™ glass microscope slides. Hematoxylin and eosin staining sections were used to identify various tissue types and the morphologic changes of tumor area. Immunohistochemical staining using specific antibodies (Beijing Zhong Shan Golden Bridge Biological Technology, Beijing, China) such as TTF-1 (ZM-0270, Clone SPT24), Napsin-A (ZM-0473, Clone IP64), p63 (ZM-0406, Clone UMAB4), p40 (ZM-0472, Clone BC28), and CK5/6 (ZM-0313, Clone OTI1F8) were used for further identification of tumor types. The histopathological diagnosis was made according to WHO criteria, and the TNM stage was determined according to the 8th edition of the Tumor, Node, and Metastasis classification of the International Union Against Cancer.

### 2.3 CTC isolation and immunostaining

5 mL blood sample was mixed with 5 mL phosphate buffered saline (PBS) and 10 mL Ficoll solution (Solarbio, Beijing, China). The mixture was centrifuged at 800×*g* for 30 min. The isolated human peripheral blood mononuclear cells layer was collected and washed with PBS at 1:1 ratio, followed by a 10 min centrifugation at 120×*g*. The supernatant was discarded, and then 10 mL of PBS was added to resuspend the human peripheral blood mononuclear cells. The solution was passed through the Labyrinth microfluidic chip, utilizing a syringe pump (PHD Ultra, Harvard Apparatus, Holliston, MA, USA). The outflow solutions containing CTCs were collected and combined with PBS to achieve a total volume of 10 mL and reinjected for a second round of separation. Finally, the outflow solutions were collected and centrifuged at 800×*g* for 10 min and supernatant was discarded. The enriched cells were fixed and loaded onto a slide, then incubated at 39°C for 30 min.

The slide was then blocked with 10% goat serum (Tianhang Biological Technology, Hangzhou, China), followed by immunofluorescence staining. Briefly, an antibody against CD45 and a pan antibody cocktail against cytokeratin (pan-CK) were added and incubated at 4°C overnight as primary antibodies (Bio-Rad, Hercules, CA, USA). Secondary antibodies conjugated with Alexa Fluor 488 and Alexa Fluor 546 were added (Life Technologies, Carlsbad, CA, USA) and incubated in dark for 45 min. Finally, a drop of mounting medium with 4′,6-diamidino-2-phenylindole (DAPI) was added (Abcam, Boston, MA, USA) and coverslips were mounted onto the slide.

### 2.4 CTC identification

The Immunofluorescence stained slides were scanned under the KF-FL-005 digital pathology scanner (KFBio, Ningbo, China) at 40× magnification. Compared to typical white blood cells from the slide with low cytokeratin expression, high CD45 expression and DAPI positive (pan-CK-/CD45+/DAPI+), cells with significantly higher cytokeratin expression, low CD45 expression and DAPI positive were identified as CTCs (pan-CK+/CD45-/DAPI+). CTCs were labeled and enumerated.

### 2.5 Statistical analysis

Ordinary one-way ANOVA (Kruskal-Wallis test) test was used to analyze the CTCs counts between all four patient groups including LC patients, BLD patients, LDCT- patient and HV. Kolmogorov–Smirnov (KS) test and ROC curve analysis with 95% confidence interval (CI) were used to were used to analyze the correlation of CTCs counts across three distinct scenarios: (1) LC patients were compared to BLD patients among LDCT+ cases. (2) LC patients were compared to the combination of BLD patients and LDCT- patients among patients with lung nodules. (3) LC patients were compared to the combination of all three control groups for all enrolled participants. From ROC curve analysis for LDCT+ cases, Youden’s index was used to identify the optimal CTCs count cutoff value and diagnostic efficiency as previously described (21). This threshold was then applied to classify patients as CTC positive or CTC negative within each group. Sensitivity, specificity and accuracy were calculated for all three distinct scenarios mentioned above. Additionally, for LC patients, the correlation of CTC counts and TNM stage were analyzed by KS test. The correlation between CTC detection and patients’ characteristics were investigated by Chi-square analyses. Moreover, correlation of lung nodule size and CTC counts was investigated using nonparametric spearman correlation, with a two-tailed p value at a 95% confidence interval. All statistical analyses and statistical graphs were conducted and generated using GraphPad Prism 9.0 software (GraphPad, San Diego, CA, USA).

## 3 Results

### 3.1 Characteristics of the study objects

The basic information of gender, age, as well as lung nodule size and locations for 167 patients with lung nodules were included in **Table 1**. Among the 113 LDCT+ patients, 52 are male, and 61 are female. For all 167 patients with lung nodules, 76 are male, and 91 are female. The median age for the patients is 58 and 56, ranging from 27-87 and 15-87 for LDCT+ patients and patients with lung nodules, respectively. Among LDCT+ patients, 41 of 113 patients have lung nodules ranging from 5mm to 10mm, whereas the remaining 72 patients exhibit nodules larger than 10mm. Among patients with lung nodules, 45 of 167 patients had small lung nodules measuring less than 5mm, while 50 patients had lung nodules ranging from 5mm to 10mm, and the remaining 72 patients had lung nodules larger than 10mm. In 6 out of the 113 LDCT+ patients, lung nodules are dispersed across more than one location, while the rest are in a single location. No lung nodule location information for LDCT- patients was collected. The pathological diagnosis shows that 96 of 113 LDCT+ patients have been diagnosed as LC, while the remaining 17 have been identified as BLD.

**Table 1:** Characteristics of patients and its correlation with CTC detection.

		LDCT+ Patients (%)	CTC+ (%)	p-Value	Patients with Lung Nodules (%)	CTC+ (%)	p-Value
Patients No.		113 (100)	84 (74.34)		167 (100)	91 (54.49)	
Age	<55 year	40 (35.40)	28 (70.00)	0.763	73 (43.71)	32 (43.84)	0.182
	≥55 year	73 (64.60)	56 (76.71)		94 (56.29)	59 (62.77)	
Gender	Male	52 (46.02)	32 (61.54)	0.266	76 (45.51)	38 (50.00)	0.562
	Female	61 (53.98)	52 (85.25)		91 (54.49)	53 (58.24)	
Lung Nodule Size	< 5 mm	–	–		45 (26.95)	5 (11.11)	NA^a^
	5≤ size ≤ 10 mm	41 (36.28)	27 (65.85)	0.546	50 (29.94)	29 (58.00)	
	> 10 mm	72 (63.72)	57 (79.17)		72 (43.11)	57 (79.17)	
Location of Lung Nodule	Upper Left	27 (23.89)	21 (77.78)	0.991	NA^b^	NA^b^	NA^b^
	Upper Right	32 (28.32)	24 (75.00)		NA^b^	NA^b^	
	Down Left	20 (17.70)	13 (65.00)		NA^b^	NA^b^	
	Down Right	17 (15.04)	15 (88.23)		NA^b^	NA^b^	
	Middle	11 (9.73)	7 (63.63)		NA^b^	NA^b^	
	≥ 1 Location	6 (5.31)	4 (66.67)		NA^b^	NA^b^	

^a^The p-Value cannot be calculated due to the selection of lung nodule size in the LDCT- group; ^b^Information regarding location of lung nodules in LDCT- patients wasn’t collected.

### 3.2 CTCs isolation and identification

**Figure 2:**
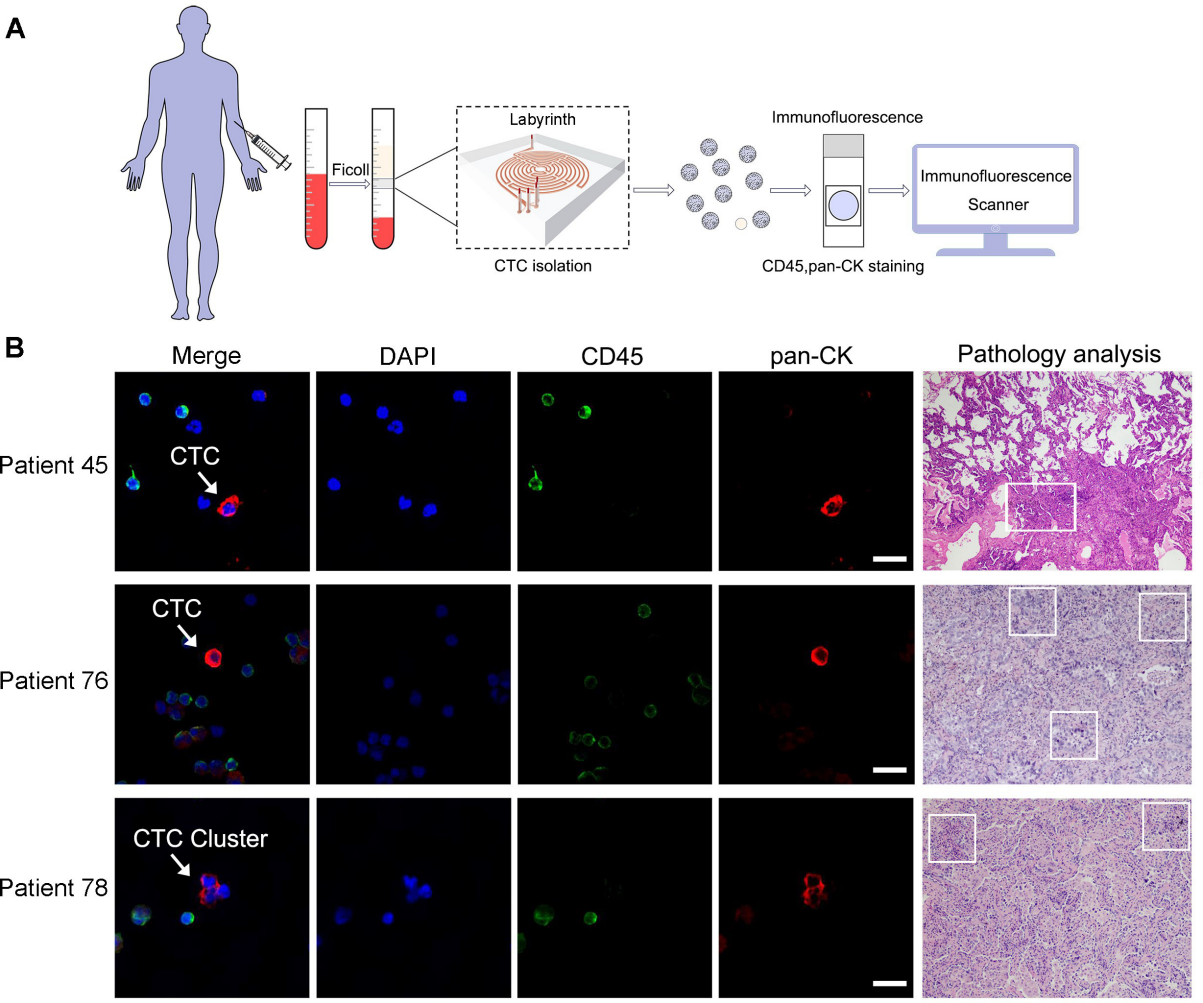
CTCs isolation and identification by Labyrinth system. **(A)** Procedures for CTCs detection from enrolled participants by Labyrinth system. **(B)** Representative views of CTCs and their corresponding pathological results from the same LC patients were obtained. CTCs from patient 45 and patient 76, as well as a CTC cluster from patient 78, were stained using DAPI, pan-CK, and an antibody against CD45. DAPI is on blue channel. The antibody against CD45 is on green channel and pan-CK is on red channel. The highlighted CTC and CTC clusters are pan-CK+/CD45-/DAPI+. White blood cells are pan-CK-/CD45+/DAPI+. Scale bar represents 10µm. Box areas in the patient’s pathological results indicate the presence of LC.

The procedures for CTC isolation and identification were summarized in **Figure 2A**. The representative view of CTCs and a CTC cluster from three LC patients, along with their pathological results, is shown in **Figure 2B**. White blood cells were identified as pan-CK-/CD45+/DAPI+ while CTCs and CTC clusters were pan-CK+/CD45-/DAPI+. The total number of identified CTCs from a 5mL blood sample was enumerated and reported as CTCs per milliliter.

Among the 96 LC patients, 5 had at least one observed CTC cluster, while no clusters were detected in BLD, LDCT-, or HV groups. In one patient, as many as 3 CTC clusters were identified. CTC clusters, which consist of more than two CTC cells, suggest increased metastatic potential and more aggressive tumor behavior. Given the rarity and significance of CTC clusters, the CTC count per milliliter for these 5 patients was reported as the sum of CTC clusters counts and the CTC counts per milliliter.

### 3.3 CTC counts and correlation to clinical and pathological characteristics

CTC enumeration was performed for all four distinct study groups, each characterized by unique clinical features. The pathological positive group, diagnosed with lung cancer as LC patient group, was distinguished from the remaining three groups, which were categorized as the non-lung cancer group.

**Figure 3:**
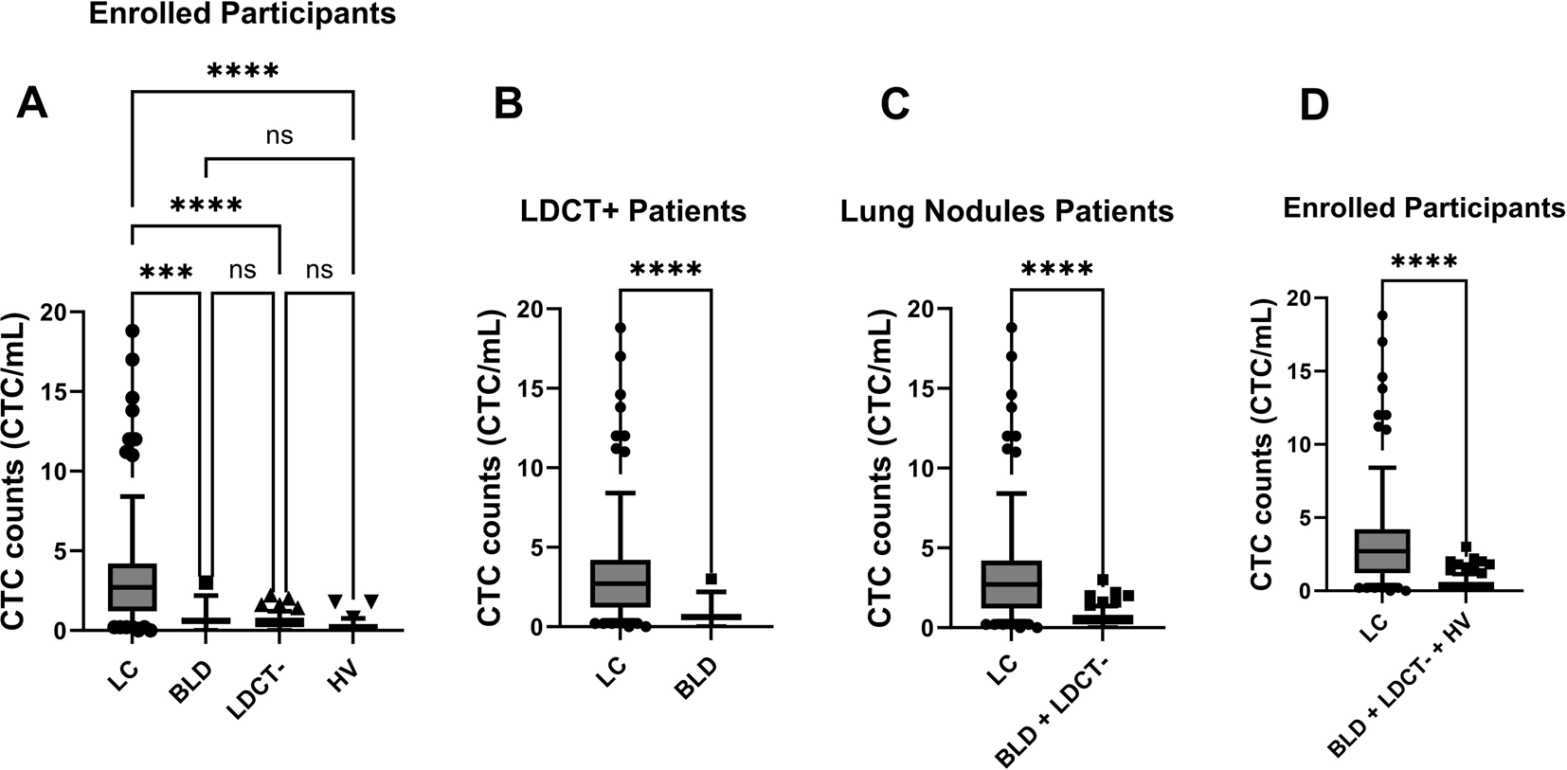
Enumeration of CTCs distinguished LC patients from control groups (BLD, LDCT-, and HV) through statistical analysis. Box-and-whiskers plots at 10-90% were used for figures. p-Values were displayed (***: p-Value < 0.001; ****: p-Value < 0.0001; ns: p-Value > 0.05). **(A)** CTC counts were compared among all enrolled participants across four distinct groups: LC patients, BLD patients, LDCT- patients, and HV. An ordinary one-way ANOVA test was used for analysis. **(B)** Among LDCT+ patients, CTC counts in LC patients and BLD patients were assessed by the KS test. **(C)** Among patients with lung nodule, CTC counts in LC patients were compared to the control group (BLD patients and LDCT- patients) using the KS test. **(D)** CTC counts in LC patients were compared to the control group (BLD patients, LDCT- patients and HV) for all enrolled participants using the KS test. The box represents the middle 50% of observed values. The bottom of the box is the first quartile (25th percentile) and the top of the box is the third quartile (75th percentile). The line in the middle of the box is the median (50th percentile). The whiskers are drawn down to the 10th percentile and up to the 90th. Points below and above the whiskers are drawn as individual dots.

The CTC counts from all four groups were plotted in **Figure 3A**. Ordinary one way ANOVA analysis was conducted to investigate the relationships between CTC counts and the four groups, with the corresponding p-values also displayed in **Figure 3A**. Significant differences between the LC patient group and the other three groups were observed with all p-Values <0.001. Between those three non-lung cancer groups, no differences were observed, which all exhibit statistical p-Values > 0.05.

To better understand how this CTC method can assist in diagnosing various scenarios, further detailed analyses were carried out. Among LDCT+ patients, including both LC and BLD groups, the CTC counts for LC and BLD patients were plotted in **Figure 3B**. For patients with lung nodules (LC, BLD, and LDCT- groups), the CTC counts from LC patients were plotted against the combined control group of BLD and LDCT- patients in **Figure 3C**. When all participants (LC, BLD, LDCT-, and HV groups) were considered, the CTC counts from LC patients were plotted against the control group, which included BLD, LDCT-, and HV patients, in **Figure 3D**. KS tests were performed for **Figures 3B–D**, and in all three scenarios, the resulting p-values were well below 0.0001, indicating significant differences in each case.

**Figure 4:**
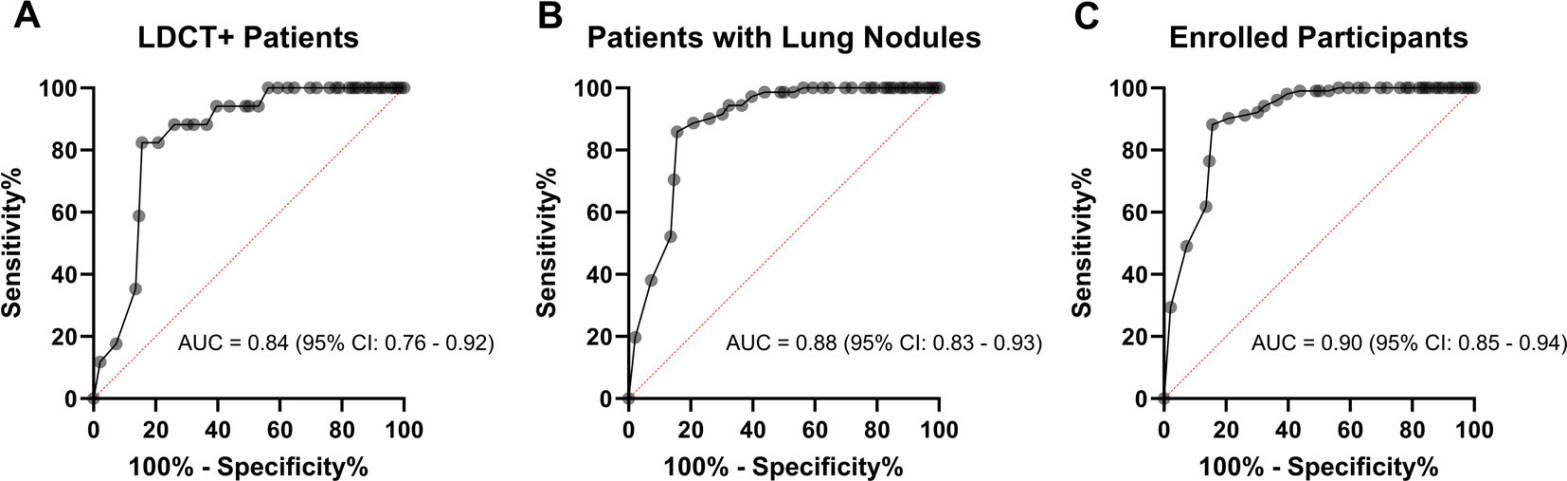
ROC curve evaluating the diagnostic potential of CTCs detected by Labyrinth system. **(A)** ROC curve comparing LC patients to BLD patients among LDCT+ patients, with displayed AUC and CI. **(B)** ROC curve for patients with lung nodules, comparing LC patients to the control group (BLD patients and LDCT- patients), with displayed AUC and CI. **(C)** ROC curve for enrolled participants, comparing LC patients to the control group (BLD patients, LDCT- patients, and HV), with displayed AUC and CI.

The ROC curve analysis was based on the data plotted in **Figures 3B–D** as well. In **Figure 4A**, the area under curve (AUC) is 0.84 (95% CI: 0.76-0.92) among LDCT+ patients. The AUC increased to 0.88 (95% CI: 0.83-0.93) among patients with lung nodules, as presented in **Figure 4B**. Furthermore, an even higher AUC of 0.90 (95% CI: 0.85-0.94) was observed for all enrolled participants, as shown in **Figure 4C**.

CTCs cut off value (≥1 CTCs/mL) was determined using Youden’s index from ROC curve analysis for LDCT+ patients. Youden’s index is a measure used to determine the optimal cutoff point on a ROC curve that maximizes the difference between the true positive rate (sensitivity) and the false positive rate (1-specificity). This helps to identify the threshold that best separates positive from negative cases. Patients were then classified as either CTC+ or CTC- based on their CTC counts. Among the cohort of 96 LC patients, 81 individuals tested CTC+, yielding a detection rate of 84.4%. The remaining 15 of the 96 patients tested CTC- and were categorized as false negatives.

For the 17 BLD patients, 14 tested CTC-, resulting in a false positive detection rate of 17.6%. Similarly, false positives were observed in both the LCDT- patients and HV, with false positive detection rates of 13.0% and 6.5%, respectively.

Drawing from the outcomes described above, the sensitivity and specificity of the CTC detection in 113 LDCT+ patients are noted as 84.4% and 82.4%, respectively. The accuracy of CTC detection for LC is 85.4%, comparable to the accuracy of LDCT diagnosis for LC, which stands at 85.0% (96/113). The sensitivity and specificity values of the CTC detection strategy increased to 84.4% and 85.9%, respectively for all patients with lung nodules. For all enrolled participants, the sensitivity is 84.4% and the specificity is 88.2%.

**Figure 5:**
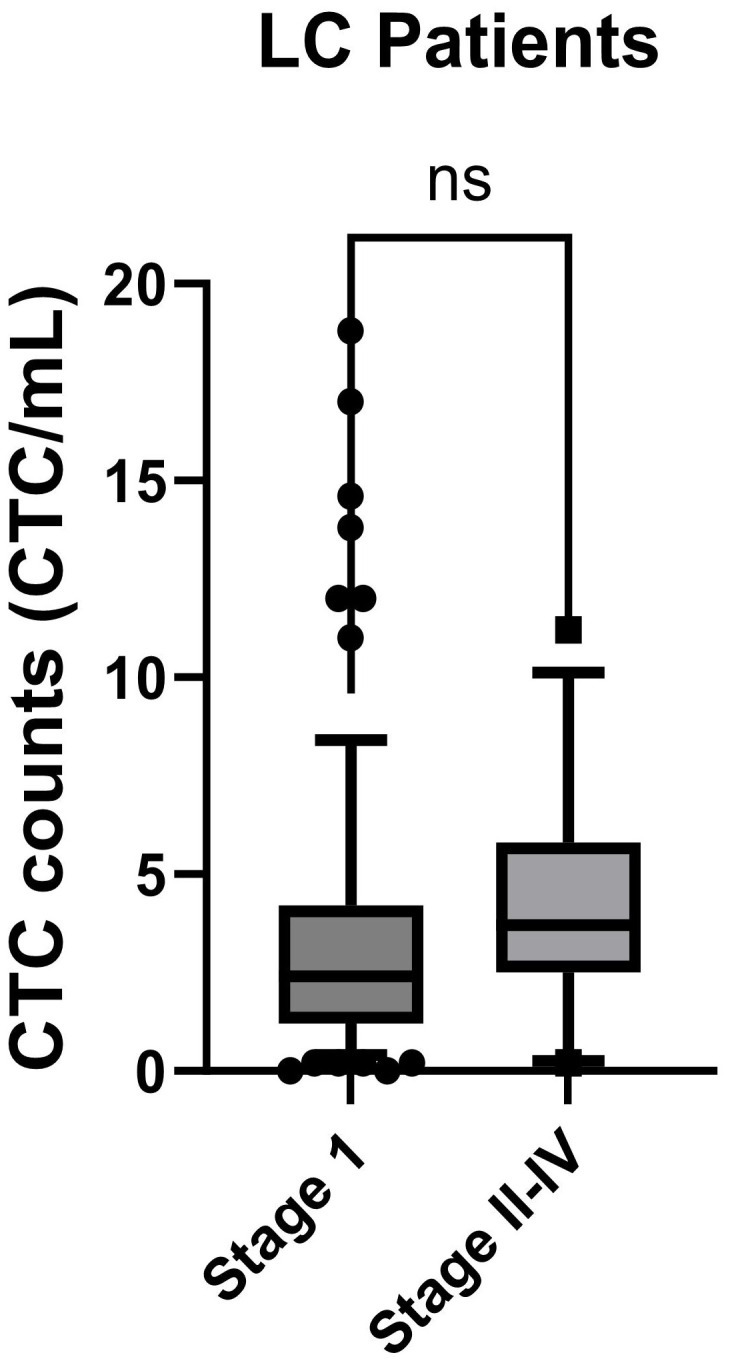
CTC counts for LC patients at different TMN stages were compared. Stage I patients were compared to the stage II-IV patients and analyzed using the KS test. Box-and-whiskers plots at 10-90% were used for the figure. p-Value was displayed (ns: p-Value > 0.05).

We extended our investigation to explore the correlation between CTC counts and the progression of LC in patients, as defined by TNM stages. Among the LC patients, 84 out of 96 were in early-stage I, while 12 out of 96 were in stage II to stage IV. The CTC counts obtained from stage I and stage II-IV were depicted in **Figure 5**. However, the p-Value of 0.27 from the KS test suggests the absence of statistically significant differences in CTC counts between the early-stage I and the later stages II-IV of LC.

**Figure 6:**
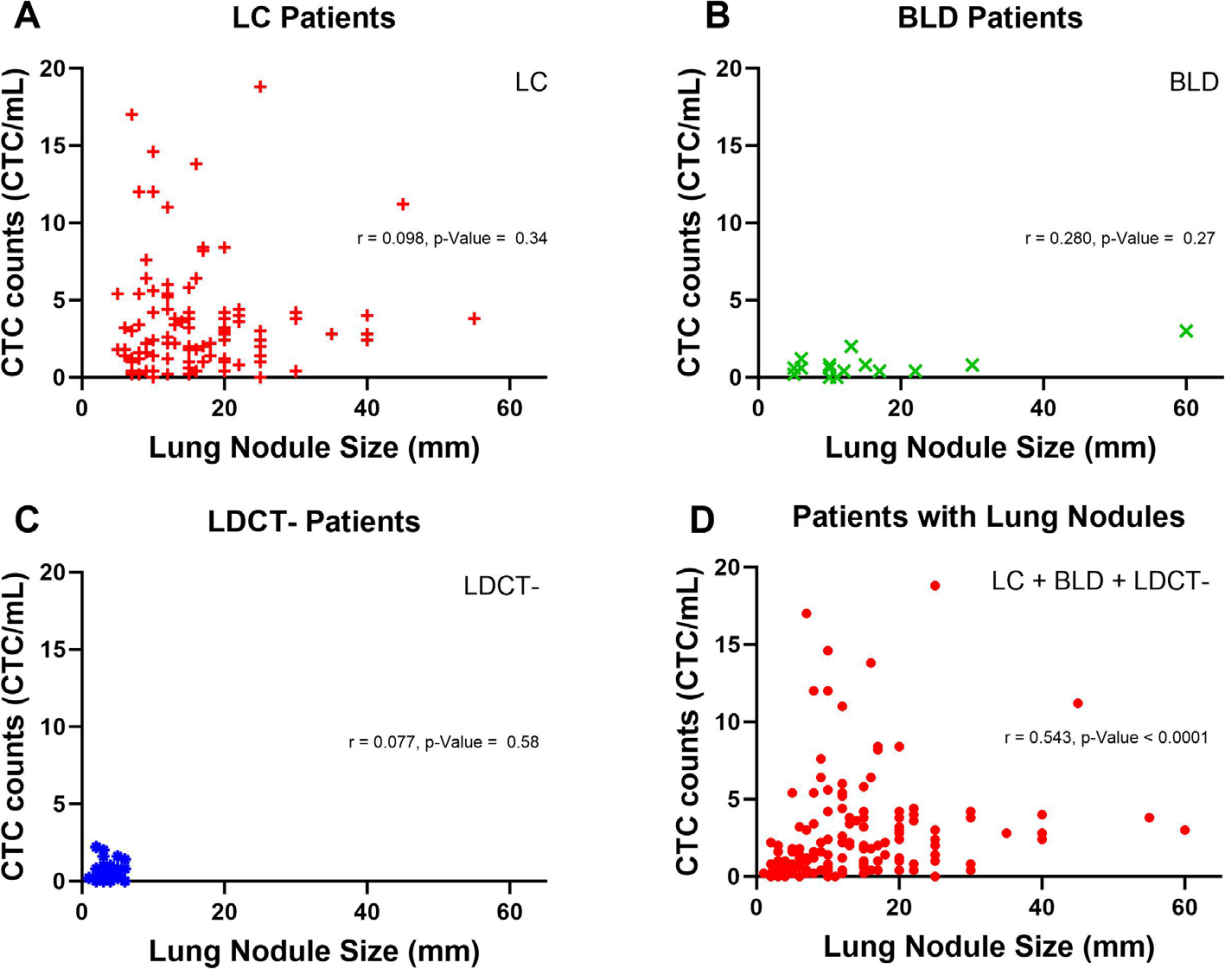
Correlation between lung nodule size and CTC counts in patients with lung nodules. **(A)** Lung nodule size and CTC counts in LC patients are represented by red plus signs. A nonparametric spearman correlation was performed to assess the relationship between lung nodule size and CTC counts for LC patients, with the correlation coefficient (r) and p-Value displayed. **(B)** In BLD patients, lung nodule size and CTC counts are shown as green crosses. A nonparametric spearman correlation was similarly conducted, with r and p-value provided. **(C)** For LDCT- patients, green crosses represent lung nodule size and CTC counts, analyzed via nonparametric spearman correlation, with r and p-value displayed. **(D)** Lung nodule size and CTC counts for all patients with lung nodules, represented by red dots, were analyzed using a nonparametric spearman correlation, with r and p-value shown.

We pursued further investigation to delve into the potential correlation between lung nodule size and CTC counts by nonparametric spearman correlation.CTC counts from patients with lung nodules were plotted against nodule size, measured by the largest diameter of one or more nodules in each patient. In **Figure 6A**, among LC patients, no correlation between lung nodule size and CTC counts was observed, with a spearman r and p-Value of 0.098 and 0.34. Similarly, no correlation was found for BLD and LDCT- patients, with p-Values of 0.27 and 0.58, respectively in **Figures 6B, C**. However, when all patients with lung nodules, including LC, BLD and LDCT- groups were analyzed together in **Figure 6D**, the result indicated a modest correlation between lung nodule size and CTC counts, yielding a spearman r value of 0.543 and a p-Value < 0.0001.

### 3.4 Correlations between CTCs and patients characteristics

The previously established CTC counts threshold (≥1 CTC/mL) was employed to categorize patients as CTC+. Chi-square analyses have been employed to explore the relationship between CTC detection and characteristics of LDCT+ patients and patients with lung nodules. This included factors such as gender, age, as well as the size and locations of the lung nodules. As detailed in **Table 1**, the p-Value for age is 0.763 and 0.182 for LDCT+ patients and patients with lung nodules, respectively. The p-Value for gender is 0.266 and 0.562 for LDCT+ patients and patients with lung nodules, respectively. Regarding lung nodule size and locations for LDCT+ patients, the p-Value is 0.546 and 0.991 respectively. Each of these p-Values for age, gender, lung nodule size, and location indicate no significant correlation observed between CTC detection and patient characteristics.

## 4 Discussion

Liquid biopsy is emerging as the most preferable non-invasive promising method for LC detection and diagnosis through biomarkers (22). Among the biomarkers, CTCs had a better diagnostic performance than traditional serum-based ones (7). Efficient isolation of CTCs is essential for the successful application of this method. Numerous methodologies have been applied to isolate and identify CTCs, each yielding varying levels of success. Additionally, SCLC typically exhibits CTC counts approximately ten times higher than those found in NSCLC (8, 13, 23, 24). In our study, the observations primarily centered on cases of NSCLC. Except for one patient whose tumor classification remained unresolved, all other diagnosed cases indisputably belonged to the NSCLC category.

Based on the ≥1 CTCs/mL cut off value derived from Youden’s index method, our CTCs detection results by Labyrinth system revealed an accuracy of 85.4%, sensitivity of 84.4%, and specificity of 82.4% among LDCT+ patients. The specificity slightly increases to 85.9% for patients with lung nodules. The occurrence of false positive results could potentially be due to either the inappropriate expression of certain CTC markers in non-cancerous cells or non-specific antibody binding to non-tumor, non-epithelial cells (25–28). The CTC detection using Labyrinth system demonstrates comparable diagnostic accuracy for LC. Research has shown false positive rates of 9.6-28.9% in LC patient diagnoses using LDCT (29). Additionally, Chest X-rays fail to identify LC in approximately 20-25% of cases (30).

Other similar studies of early-stage LC detection by CTCs have been carried out by several different CTCs isolation methods. EpCAM dependent CTC isolation methods from both Cellsearch and CellCollector have been evaluated with low sensitivity and selectivity (31, 32). ISET method for CTC isolation and detection is not suitable for LC screen (33). CTCs detected by folate receptors showed 70% stage I sensitivity for NSCLC (34). However, a study focusing on advanced-stage LC patients, utilizing size based filters to isolate CTCs in microfluidic chips, had reported 82% sensitivity and 100% specificity in LC patients (35). These findings underscore the need for optimizing epitope-independent strategies for CTC isolation (36). Consequently, Labyrinth’s system, specifically designed for CTC isolation based on cell size, presents a promising solution. In this study, the Labyrinth system demonstrated 84.4% sensitivity and 82.4% specificity for LDCT+ patients, with 84 out of 96 LC patients were in stage I, underscoring its potential suitability for early-stage LC detection and diagnosis.

Additionally, we were able to capture CTC clusters from LC patients. Notably, a stage IV patient exhibited the highest count of CTC clusters in this study. The presence of CTC clusters is acknowledged as a biomarker in advanced-stage cancer patients (37). The detection of CTC clusters has the potential to assist in reevaluating the results of clinical diagnosis.

In this study, we did not find a significant correlation between CTCs count and LC stages. Previous investigations from various research groups have yielded conflicting results regarding this correlation. Studies using EpCAM or ISET methods found no correlation between CTC counts and LC TMN stage (38–40). In contrast, those employing size based filters or negative enrichment methods reported increased CTC counts in stage IV NSCLC patients compared to other stages (35, 41). Variations in CTC isolation methods may contribute to the observed disparities. Additionally, the limited sample size, with only 12 out of 96 patients in stages II to stage IV, may have impacted our ability to detect correlations in advanced lung cancer stages.

Furthermore, no correlation was observed between CTC counts and lung nodule size within the individual groups of LC, BLD, and LDCT-. In the non-lung cancer groups of BLD and LDCT-, the absence of correlation is expected. In the LC groups, the lack of correlation between CTC counts and lung nodule size may be attributed to the limited sample size in advanced lung cancer stages and the predominance of lung nodules measuring less than 20 mm. However, when all patients with lung nodules were analyzed together, a weak correlation between CTC counts and lung nodule size was observed in our study. This finding is relevant, especially considering the established association between larger lung nodules and increased lung cancer risk (42).

One notable limitation of the current study is that it was conducted at a single location, the First Affiliated Hospital of USTC in Anhui, China. This single-site selection may introduce biases in patient selection. While the results are promising, conducting a multi-site study with a larger number of enrolled patients could enhance the robustness and generalizability of the findings. Additionally, the limited patient number from this site may impact the implications of false positives and negatives in clinical settings. A broader patient population may provide more diverse data, allowing for a more comprehensive evaluation of the Labyrinth system’s efficacy in CTC detection across different demographics and clinical settings.

In summary, findings from the study highlight the potential of employing Labyrinth system as a valuable tool for utilizing CTCs as a biomarker in the early detection and diagnosis of LC. The high sensitivity and specificity of this method facilitates the application of CTCs in the diagnosis of early-stage LC in clinical practice.

## Data Availability

The original contributions presented in the study are included in the article/supplementary material. Further inquiries can be directed to the corresponding authors.
